# Intracellular Doppler Signatures of Platinum Sensitivity Captured by Biodynamic Profiling in Ovarian Xenografts

**DOI:** 10.1038/srep18821

**Published:** 2016-01-06

**Authors:** Daniel Merrill, Ran An, Hao Sun, Bakhtiyor Yakubov, Daniela Matei, John Turek, David Nolte

**Affiliations:** 1 Department of Physics, Purdue University, West Lafayette, Indiana; 2 Department of Basic Medical Sciences, Purdue University, West Lafayette, Indiana; 3 Department of Medicine, Indiana University School of Medicine, Indianapolis, Indiana; 4 Indiana University Simon Cancer Center, Roudebush VA Hospital, Indianapolis, Indiana; 5 Animated Dynamics, Inc., West Lafayette, Indiana

## Abstract

Three-dimensional (3D) tissue cultures are replacing conventional two-dimensional (2D) cultures for applications in cancer drug development. However, direct comparisons of *in vitro* 3D models relative to *in vivo* models derived from the same cell lines have not been reported because of the lack of sensitive optical probes that can extract high-content information from deep inside living tissue. Here we report the use of biodynamic imaging (BDI) to measure response to platinum in 3D living tissue. BDI combines low-coherence digital holography with intracellular Doppler spectroscopy to study tumor drug response. Human ovarian cancer cell lines were grown either *in vitro* as 3D multicellular monoculture spheroids or as xenografts in nude mice. Fragments of xenografts grown *in vivo* in nude mice from a platinum-sensitive human ovarian cell line showed rapid and dramatic signatures of induced cell death when exposed to platinum *ex vivo*, while the corresponding 3D multicellular spheroids grown *in vitro* showed negligible response. The differences in drug response between *in vivo* and *in vitro* growth have important implications for predicting chemotherapeutic response using tumor biopsies from patients or patient-derived xenografts.

Current methodologies used to predict response to therapy rely on chemosensitivity assays that test patient-derived cancer cells to chemotherapy^1^,^2^. These culture assays have limited ability to test cancer cells from clinical specimens, lack predictive power for subsequent clinical applications^3^,^4^,^5^, and rely on epithelial tumor components. It is known that cells grown in 2D respond differently to therapeutic agents compared to cells in 3D tissues, displaying different genetic expression profiles^6^,^7^,^8^, possessing distinct intercellular signaling^9^, and responding to different forces exerted from their environment^10^. Of particular importance, cellular dimensionality and microenvironment exert an essential influence on the drug sensitivity^11^,^12^,^13^ of 3D samples. Therefore, cell-based sensitivity assays are transitioning from 2D to 3D formats that represent a more natural cell environment^14^,^15^ and allow formation of cellular contacts to the extracellular matrix and to other cells. These modulate intracellular signaling^8^,^16^ and gene expression^6^,^7^,^17^, more closely replicating the tumor microenvironment. Examples of 3D tissue models that are used to study tumor response to treatment include patient-derived xenografts (PDX)^18^, multicellular spheroids^18^ and patient-derived organoids^19^. In these models, tumor heterogeneity and spatially diverse microenvironments remain a challenge, raising the need for novel imaging approaches that can map spatially-varying tumor response to treatment.

Biodynamic imaging (BDI) is a deep three-dimensional optical imaging approach with sensitivity to cellular motions^20^ that yields specific signatures for dynamic cellular functions^21^. BDI uses coherence-gated digital holography to optically “section” *in vitro* tissue up to 1 mm deep^22^ as a full-frame imaging approach closely related to *en face* optical coherence tomography^23^. Digital holography^24^,^25^,^26^,^27^ uses a CCD camera to capture a digital Fourier off-axis hologram and numerically reconstruct an image of the tissue. BDI is sensitive to intracellular motions through Doppler light scattering with three decades of dynamic range across frequencies from 0.01 Hz to 12.5 Hz, responding to organelle and vesicle motion driven by molecular motors, to cytoplasmic streaming and restructuring, to cytoskeletal forces and to membrane modulation^28^. One of the imaging formats of BDI is called tissue-dynamics spectroscopy (TDS) that provides functional imaging by analyzing fluctuating speckle-intensity time-series into individual ultra-low frequency (ULF) Doppler components. Different frequencies relate to different types of motion, and TDS can time-resolve changes in these motions as tissues react to environmental or pharmacological perturbations^29^. We previously validated BDI in applications of drug screening and phenotypic profiling^30^, but this is the first application of the technique to pre-clinical cancer biology. Ovarian cancer was chosen as a model, because most patients relapse after first-line platinum and taxane-based chemotherapy, and development of a methodology able to predict initial or subsequent response to treatment would be useful for selecting chemotherapeutic or biological agents most likely to arrest tumor growth. Distinction between platinum resistant and sensitive phenotypes is well studied biologically and is highly relevant clinically.

The biodynamic imaging system (Fig. 1a) combines low-coherence infrared backscattering with digital holography. The coherence length of the super-luminescent light source is approximately 20 microns. Polarized light is backscattered from the tumor sample and directed through Fourier transform lens pairs to the digital pixel-array that resides on an optical Fourier plane. The digital hologram is reconstructed using a fast Fourier transform. In living tissue, all the constituents of cells are in motion, producing Doppler frequency shifts in the scattered light proportional to the longitudinal speed of the scattering particle 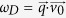 , where *q* is the momentum transfer and *v*_*0*_ is the particle velocity (Fig. 1b). The frequency range for biodynamic imaging is 0.01 Hz to 12.5 Hz corresponding to Doppler speeds of 2 nm/sec to 2 microns/sec (Fig. 1c). All intracellular motions are driven by active transport, supported by ATP or GTP metabolism that drives molecular motors, membrane fluctuations, cytoskeletal reconstruction and subsequent membrane shape changes (Fig. 1d). There are no detectable thermal diffusion or Brownian motion signatures in our detection frequency range. Doppler spectroscopy of intracellular motions is broadband because intracellular motions in 3D are isotropic, and directed motions have finite correlation (or persistence) time t_0_. These characteristics produce an ensemble of Rayleigh flights (rather than a Wiener process^31^) characterized by a mean-squared displacement (MSD) 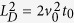 that produces a one-sided generalized Lorentzian power spectrum 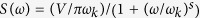 with variance V, a knee frequency 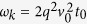 and a slope parameter *s*. Distinct physiological processes produce multiple spectra that superpose into a broadband spectrum with multiple knee frequencies ω_k_ (Fig. 1c). Applied therapeutics modify the individual spectra differently, producing distinct fingerprints of the modified physiological effects^30^.

## Results

### Biodynamic imaging of tumor spheroids and xenograft fragments

**Figure 1:**
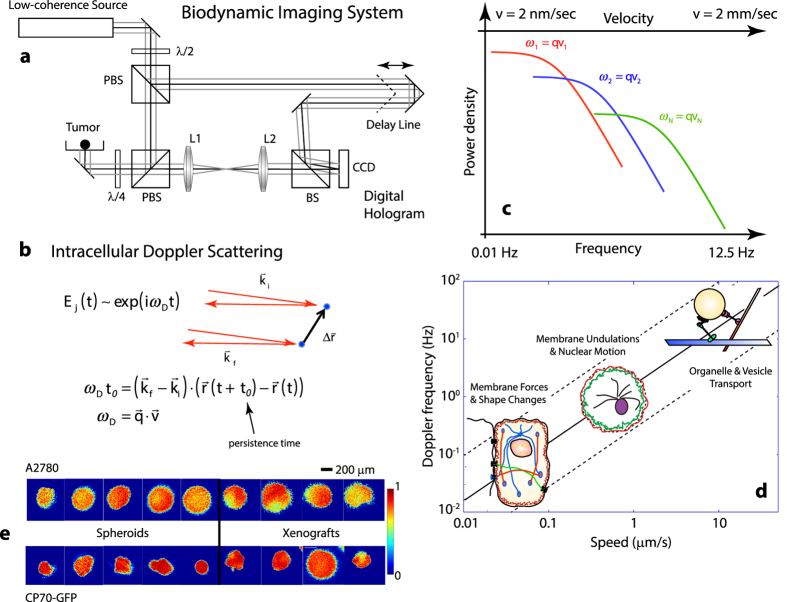
Principles of biodynamic imaging. (**a**) The biodynamic imaging system with a low-coherence light source, backscattering from the tumor sample and detection using digital holography on the Fourier plane. (**b**) Intracellular light scattering generates a Doppler frequency shift caused by active transport with speed v and persistence time t_0_. (**c**) Schematic illustrating frequency and speed ranges for several Doppler knee frequencies that correspond to the three basic physiological motions depicted in (**d**). (**e**) Motility contrast images of *in vitro* tumor spheroids and *ex vivo* xenograft biopsies grown from A2780 and A2780/CP70 cell lines.

**Figure 2:**
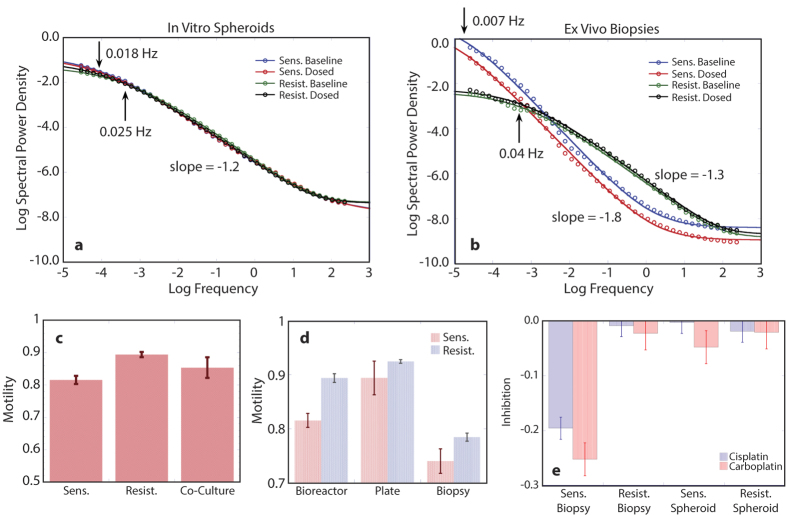
Biodynamic imaging data for sensitive and resistant cell lines. (**a**) Spectral power density pre- and post-dose for spheroids of sensitive (A2780) and resistant (A2780/CP70) cell lines treated with cisplatin and averaged over all samples (n = 11 for A2780 and n = 13 for A2780/CP70). Neither cell line shows significant alterations in power spectrum shape or NSD after exposure to cisplatin. (**b**) Spectral power density pre- and post-dose for *ex vivo* biopsies for the sensitive and resistant cell lines treated *ex vivo*. (**c**) Average initial motility for *in vitro* spheroids of monocultures of each cell line and co-culture of both cell lines. (**d**) Average initial motility of sensitive and resistant samples for *in vitro* bioreactor, *in vitro* 96-well plate, and *in vivo* intraperitoneal tumor growth models. (**e**) Overall inhibition (ALLF) for *ex vivo* biopsies and *in vitro* spheroids treated with 10 μM cisplatin and carboplatin.

Tumor spheroids were grown in a rotating bioreactor (Synthecon) or in U-bottom spheroid plates (Corning) that provide sufficient time for cells to generate multidimensional interactions and deposit extracellular matrix as they form tissue-like aggregates morphologically similar to naturally-occurring avascular tumors grown *in vivo*. The same cell lines cultured as spheroids were used to generate intraperitoneal xenografts in nude female mice, and the millimeter-scale tumor explants were used for analysis. BDI was performed on bioreactor-grown ovarian cancer spheroids or tumor explants exposed to two platinum compounds (cisplatin and carboplatin). The results of BDI for spheroids and xenograft fragments are presented in several modalities, one of which is motility contrast imaging (MCI) shown in Fig. 1e for spheroids. MCI uses total fluctuation amplitude as the imaging contrast and produces spatial maps of overall intracellular motion. Tissue regions that are highly active are pseudo-colored red, while tissue regions with low activity are pseudo-colored blue (see Methods section). The samples derived from the platinum-resistant cell lines (A2780/CP70 or A2780cis) showed overall stronger intracellular motility than those derived from the platinum-sensitive cell line (A2780). The average intracellular motility for the sensitive tissue grown as spheroids was 0.82 ± 0.01 and for the resistant tissue was 0.91 ± 0.06. The average intracellular motility for the xenografts derived from sensitive cells was 0.74 ± 0.02 and for the resistant xenografts was 0.78 ± 0.01 (Fig. 2c,d)

Biodynamic imaging captures information accessed from the fluctuation spectral power density. Average pre- and post-dose fluctuation power spectra for the A2780 (n = 11) and A2780/CP70 (n = 24) cell lines grown as *in vitro* spheroids and responding to cisplatin (10 μM) are shown in Fig. 2a. The post-dose spectra were obtained 9 hours after treatment. Spheroids from both cell lines displayed knee frequencies around 0.02 Hz with slope parameters *s* = 1.2. The platinum compounds had only modest effect on the spectra of the spheroids. In contrast, the average pre- and post-dose fluctuation power spectra for the tumor explants are shown in Fig. 2b. The knee frequency of the resistant cell line was around 0.04 Hz with a slope parameter *s* = 1.3, while the sensitive cell line tumor fragments had a much lower knee frequency below 0.01 Hz, but with a higher slope parameter *s* = 1.8. Exposure to platinum had a strong effect on the power spectrum of the sensitive cell line. The significant differences in the fluctuation power spectra of xenografts relative to the grown spheroids reflect differences arising from the different growth environments. The overall spectral responses of the tumor fragments to 10 μM platinum exposure are shown in Fig. 2e and are compared with the responses of the same cell lines grown *in vitro* as spheroids. The tumors derived from the sensitive cell line displayed significant response to both cisplatin and carboplatin, while the resistant cell line xenografts displayed negligible response. All of the spheroids, whether from the sensitive or resistant cell lines, displayed little response to platinum over the 9-hour incubation with the agent.

### Population separation using a logistic predictor

**Figure 3:**
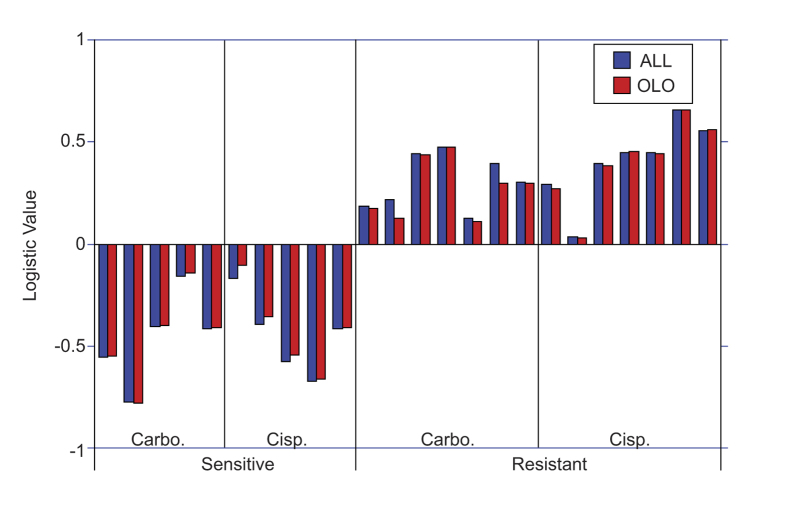
Logistic predictor model using selected biomarkers. The logistic predictor used three biomarkers (ALLF, APOP and KNEE) of 24 individual biopsy samples across sensitive (A2780) and resistant (A2780/CP70 and A2780cis) cell lines responding to 50 μM cisplatin (Cisp.) and carboplatin (Carbo.) treated *ex vivo*. Blue bars are results of training the logistic function with all samples. Red bars are results of the one-left-out (OLO) cross validation.

We propose that *ex vivo* treatment of tumors with platinum, combined with real-time monitoring of biodynamic tissue response to treatment, could be used as an assay to measure chemosensitivity or resistance of living tissue. To capture the characteristic response signatures of the *ex vivo* tissue, we define several biodynamic biomarkers that measure different aspects of the tissue response to treatment. For example, pre-condition metrics capture the baseline biodynamic behavior of the tumor tissue, while drug-response metrics capture the alterations in the biodynamic signatures of the tissue caused by the treatment. The pre-condition metric that correlates most strongly with objective response is the knee frequency of the baseline fluctuation spectrum (see Fig. 2b). The drug-response metrics that correlate strongly with objective response are the overall inhibition (see Fig. 2e) as well as a nonlinear response in the drug-response spectrograms that is representative of apoptosis (see Methods section). These three biodynamic biomarkers (designated as KNEE, ALLF, and APOP) are shown in Supplementary Fig. S1 for the tumor samples analyzed (n = 24). The biomarkers were combined using standard deviations and Kolmogorov-Smirnov thresholds into a single argument for a logistic predictor model to predict the response of each sample (see Methods section). The parameters of the logistic predictor were set using data derived from all tumors, and the results are shown as the blue bars on the graph in Fig. 3. There is a clear separation between the responses of the sensitive and the insensitive tumor types to both carboplatin and cisplatin. To test for overfitting of the data by the logistic predictor, a one-left-out (OLO) cross validation analysis was performed in which the logistic function was trained using 23 of the biopsies, holding one of the samples back that was subsequently tested in the predictor. This procedure was repeated for each of the 24 tumor fragments, and the results are shown in Fig. 3 as the red bars. The performance of the OLO assay is almost identical to the full assay, with 100% separation between the two populations. When the logistic prediction values were fit by continuous Gaussian distributions to generate a smooth a receiver-operator curve (ROC), the accuracy, sensitivity and specificity of the OLO analysis were all above 95%.

## Discussion

We demonstrate that BDI can be used for preclinical prediction of response to therapy by testing fragments of tumor xenografts for response to platinum. As expected, cell lines known to be sensitive to platinum yielded xenografts that were sensitive when tested *ex vivo*, and platinum-resistant cells generated explants that were resistant to *ex vivo* treatment with platinum. The sensitivity and accuracy of BDI and the newly-defined dynamical biomarkers was greater than 95% across 24 specimens. However, in contrast to the high-accuracy performance of the BDI chemoresponse assay on *ex vivo* tumors, we observed no significant differential response to platinum between the two cell lines grown as spheroids either in bioreactor or in U-bottom plates. These results obtained using the *in vitro* model have important ramifications. First, they highlight important differences between 3D *in vitro* and *in vivo* tissue growth. The two environments are different and outgrowth of different clonal populations can occur *in vivo*. Furthermore, tumors grown *in vivo* include stromal and vascular cells that are likely to alter response to treatment and survival of cancer cells^11^,^12^,^13^ (See Supplementary Fig. S2). Second, the negative results of BDI on spheroids derived from cell lines with well-defined sensitivity/resistance response to platinum in 2D highlight critical differences between 2D and 3D models. Drug transport and penetration in 3D culture are different than in 2D monolayers, leading to EC50 values that typically differ by an order of magnitude^28^. For instance, the use of 3D culture for chemoresponse testing requires higher drug concentrations, which consequently may induce off-target effects. These issues can confound interpretations and predictions derived from 3D culture assays. On the other hand, tumor explants display the anticipated sensitivity/resistance response to platinum and may be more predictive of therapeutic response than 3D spheroid culture. Our data support that BDI provides effective and specific 3D functional imaging of chemotherapy response, and could be further developed for applications in personalized cancer care.

## Methods

### Animal and tissue models

A2780 and A2780cis ovarian cancer cells were from Sigma. A2780/CP70 cells were a gift from Dr. Bob Sanders, University of Texas at Austin. Cells were cultured in RPMI 1640 (Cellgro, Manassas, VA) supplemented with 10% fetal bovine serum (FBS) (Cellgro) and 1% antibiotics^32^. Xenografts were generated through intraperitoneal (ip) implantation of 5 × 10^6^ cells into 6-7 week-old nude female BalbC mice. All animal experiments were approved by the Indiana University Animal Care and Use Committee, being in compliance with federal regulations. Animal experiments were carried out in accordance with approved guidelines. IP xenografts formed and were harvested after 4 weeks. Harvested tumors were placed in ice cold media and were transported to the imaging laboratory within 2 hours. Samples approximately 1 mm^3^ in volume were immobilized in multi-well plates in preparation for BDI. This extraction/transport/preparation protocol ensured that the samples experienced limited degradation during transport and preparation for BDI.

To generate 3D tissue culture, cells were grown as 3D tumor spheroids using a rotating bioreactor (Synthecon, Houston, TX) or Corning U-bottom spheroid plates. A 50 mL capacity Sythecon bioreactor was seeded with 2 × 10^6^ cells in RPMI 1640 growth medium with 2 mM glutamine and 10% fetal bovine serum. Visible spheroids (200–300 μM) were formed by 7 days. Spheroids typically had diameters between 150 and 500 microns. A2780 spheroids formed relatively tight and spherical tissues, while the A2780/CP70 spheroids were looser cellular aggregates. The U-bottom growth technique generates spheroids more rapidly by using high cell seeding density. The 96-well plates were seeded with 1–5 × 10^4^ cells and tumor spheroids formed in 48–72 hours. In general, the U-bottom samples had lower cohesion and lacked extensive cellular adhesions and minor extracellular matrix compared to the samples grown in bioreactors. The availability of these three growth approaches (*in vivo* intraperitoneal, *in vitro* bioreactor and *in vitro* plate) provides a degree of control over the tissue properties of samples deriving from the same cell line

### Biodynamic imaging

Motility contrast images of tumor spheroids grown in the bioreactor (Fig. 1e) capture the overall subcellular and cellular motions, represented in a pseudo-color scale of the normalized standard deviation and reflects the general activity of the tissue displayed in spatial maps^19^. The spatial motility map across a field of pixels is defined as:


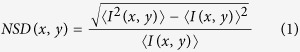


where I(x,y) is the intensity of the holographic reconstruction of the pixel at location (x,y). A Poisson fluctuation process yields NSD = 1, while long-term temporal correlations reduce the NSD values below unity. NSD values for the most energetic tissues approach unity, while less energetic tissues have lower values.

The average motility metric (NSD) values of the tumor spheroids were 0.82 ± 0.01 (n = 56) for platinum-sensitive A2780 and 0.91 ± 0.06 (n = 53) for platinum-insensitive A2780/CP70 cells. Several spheroid samples were also grown as co-cultures of both cell lines, and these had an average NSD of 0.85 ± 0.06 (n = 4), which is approximately the average of the two cell lines (Fig. 2c). A comparison with bioreactor-grown spheroids and *ex vivo* tumor explants shows that the U-bottom plate samples had the highest motility with an average NSD of 0.89 ± 0.03 (n = 8) and 0.92 ± 0.01 (n = 30) for A2780 and A2780/CP70, respectively (Fig. 2d). In general, both cell lines had lower motility when grown in the bioreactor (0.82 ± 0.01, n = 43; 0.89 ± 0.01, n = 18). The lower bioreactor motility is consistent with the presence of denser intercellular adhesions that constrain the motion of the cell membrane, which is a major contributor to the overall motility metric. Average values for A2780 and A2780cis xenografts are 0.74 ± 0.02 and 0.78 ± 0.01, respectively.

The BDI protocol for testing drug response is called tissue dynamics spectroscopy (TDS)^30^. A baseline spectrum is established over several hours, after which a drug or other perturbation is applied to the samples. Time-frequency drug-response spectrograms are generated using


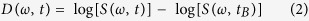


where S(ω,t) is the frequency spectrum acquired at time t, and S(ω,t_B_) is the baseline spectrum. Drug-response spectrograms capture relative changes in fluctuation power spectra as a function of time.

Examples of TDS spectrograms for positive and negative controls are shown in Supplementary Fig. S3 for the mitochondrial toxin *p*-triflouromethoxyphenylhydrazone (FCCP) and for the carrier dimethyl sulfoxide (DMSO) applied to spheroids grown *in vitro*. The response of both A2780 and A2780/CP70 to FCCP is similar to TDS spectrograms obtained on tumor spheroids from other cell lines^33^. The negative controls were relatively nonresponsive, with minor changes over time that are typical of healthy tissue samples that continue to proliferate.

### Proliferation assay

Exponentially growing A2780 and CP70 cells were seeded in 96-well plates. Twenty four hours after seeding, cells were treated with 2, 4, 8, 16, 32 uM of cisplatin, After 72 hours of drug exposure, a CCK-8 assay was performed according to manufacturer’s specifications (Dojindi). Growth inhibition curves were generated, where each point represents mean ± SD of 3 replicates. The 50% inhibitory concentration (IC_50_) value was calculated by using nonlinear regression by fitting the normalized data to a sigmoid dose–response curve (Supplementary Fig. S4). The treatment of cells cultured as spheroids was carried out using the same cisplatin concentrations for 72 hours, and normalized data were plotted. Distinct dose-dependent decreases in cell viability upon treatment with cisplatin were observed for A2780 (sensitive) and CP70 (resistant) cells, when grown as monolayers. Spheroids were significantly more resistant to cisplatin compared to 2D cultures and differences between sensitive and resistant cells were not observed (Supplementary Fig. S4).

### Tissue response to platinum compounds

The average TDS spectrograms of spheroids grown *in vitro* responding to cisplatin and carboplatin at two concentrations applied to both cell lines are shown in Supplementary Fig. S5. The drug response of the sensitive/resistant tumor spheroids to cisplatin and carboplatin showed very little change over 9 hours, and were not statistically different than the spectrograms from the DMSO negative control. There was little variability between the responses of different samples, with the standard deviation typically ranging from 0.1 to 0.2.

Average spectrograms across the two cell lines and the two platinum compounds are shown in Supplementary Fig. S6 for *ex vivo* tumors and *in vitro* spheroids. The A2780 tumors displayed strong inhibition by platinum, while the A2780/CP70 (and A2780cis for the data in the red box) tumors were resistant. The tumor spectrograms are contrasted with the spheroid spectrograms that show little difference between the two cell lines.

### Logistic drug response predictor

To define the performance of the predictive assay for therapy response, a logistic model was constructed which incorporated three biomarkers. These are apoptosis-related (APOP), all-frequency (ALLF) and knee-frequency (KNEE) motility metrics. The ALLF biomarker value is obtained through a linear filter applied to the spectrogram and integrated over log frequency.


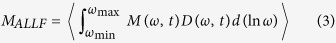


The KNEE biomarker is the knee frequency of the spectrum, and APOP is a nonlinear filter that identifies enhanced frequency bands at high and low frequencies (a signature that previously has been correlated with apoptosis^30^). These three metrics correlated most strongly with the two cell line populations (sensitive and insensitive). By combining the three motility metrics in the multivariate logistic function, the combined group predicted chemotherapy response in 100% of cases using a binary classifier (response *vs*. non-response) that fully separated the two groups (Fig. 3). The argument in the logistic function is constructed from the mean values and standard deviations of the motility metrics in Supplementary Table S1 to construct a multivariable logistic predictor of drug response as


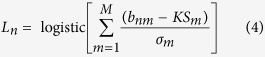


where L_n_ is the logistic drug response predictor for the n^th^ sample, m varies over the selected biomarkers, M is the number of biomarkers, b_nm_ is the value of the m^th^ biomarker for the n^th^ sample, s_m_ is the standard deviation of the m^th^ biomarker, and KS_m_ is the Kolmogorov-Smirnov threshold between the two populations (responsive and non-responsive) for the m^th^ biomarker.

## Additional Information

**How to cite this article**: Merrill, D. *et al.* Intracellular Doppler Signatures of Platinum Sensitivity Captured by Biodynamic Profiling in Ovarian Xenografts. *Sci. Rep.* **6**, 18821; doi: 10.1038/srep18821 (2016).

## Supplementary Material

Supplementary Information
