# Supplementary material for: Intracellular Doppler Signatures of Platinum Sensitivity Captured by Biodynamic Profiling in Ovarian Xenografts
Source: Sci Rep. 2016 Jan 6;6:18821. doi: 10.1038/srep18821 (PMC4702146; doi:10.1038/srep18821)
Supplement: Supplementary Information [file srep18821-s1.pdf]

Supplementary Information

Intracellular Doppler Signatures of Platinum Sensitivity Captured by Biodynamic Profiling in Ovarian Xenografts

Dan Merrill [1], Ran An [5], Hao Sun [1], Bakhtiyor Yakubov [3], Daniela Matei [3, 4], John Turek [2, 5] and David Nolte [1, 5]

[1]Department of Physics, Purdue University, West Lafayette, Indiana.
[2]Department of Basic Medical Sciences, Purdue University, West Lafayette, Indiana.
[3]Department of Medicine, Indiana University School of Medicine, Indianapolis, Indiana.
[4]Indiana University Simon Cancer Center, Roudebush VA Hospital, Indianapolis, Indiana.
[5]Animated Dynamics, Inc., West Lafayette, Indiana.

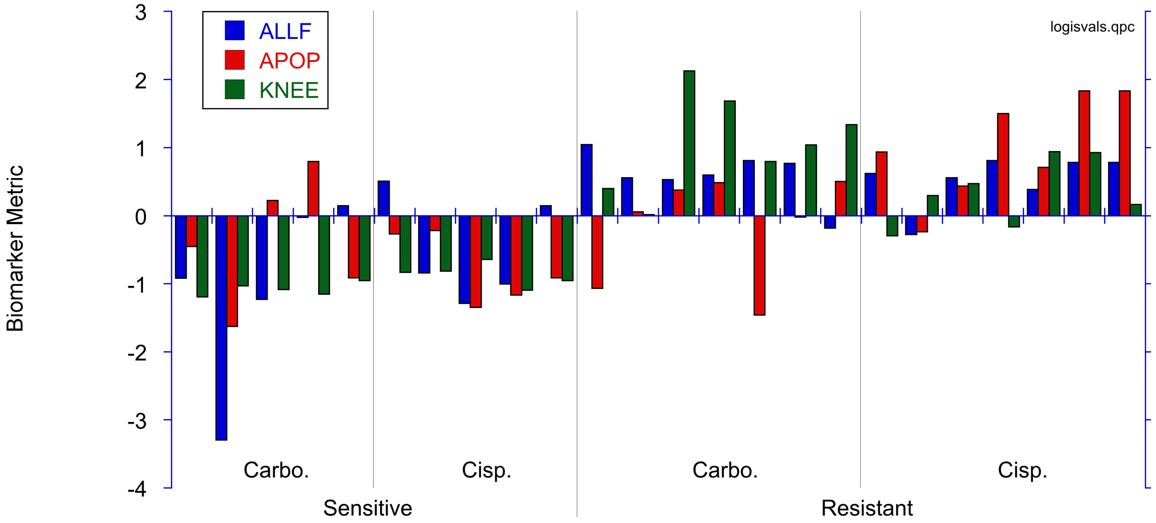

Supplementary Figure S1 Values for three biodynamic biomarkers (ALLF, APOP and KNEE) measured across 24 samples. The samples are grouped as sensitive/carboplatin, sensitive/cisplatin, insensitive/cisplatin and insensitive/carboplatin. The ALLF biomarker measures overall inhibition in the drug-response spectrogram. The APOP biomarker is a nonlinear metric that correlates with apoptotic response. The KNEE biomarker is the knee frequency of the fluctuation spectral power.

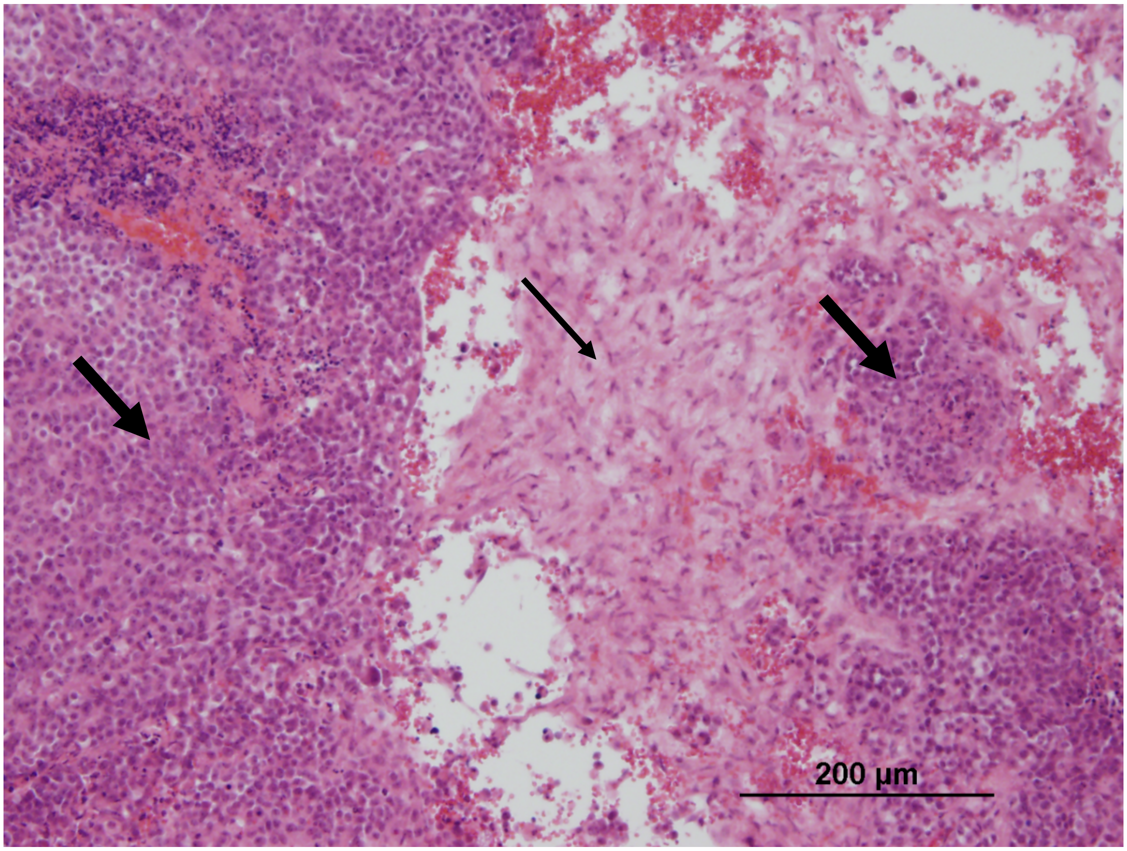

Supplementary Figure S2 Optical H&E stain thin section of A2780cis explant tumor. The heterogeneous nature of explant tumors is demonstrated by areas of stromal tissue (thin arrow) mixed with tumor tissue (large arrows).

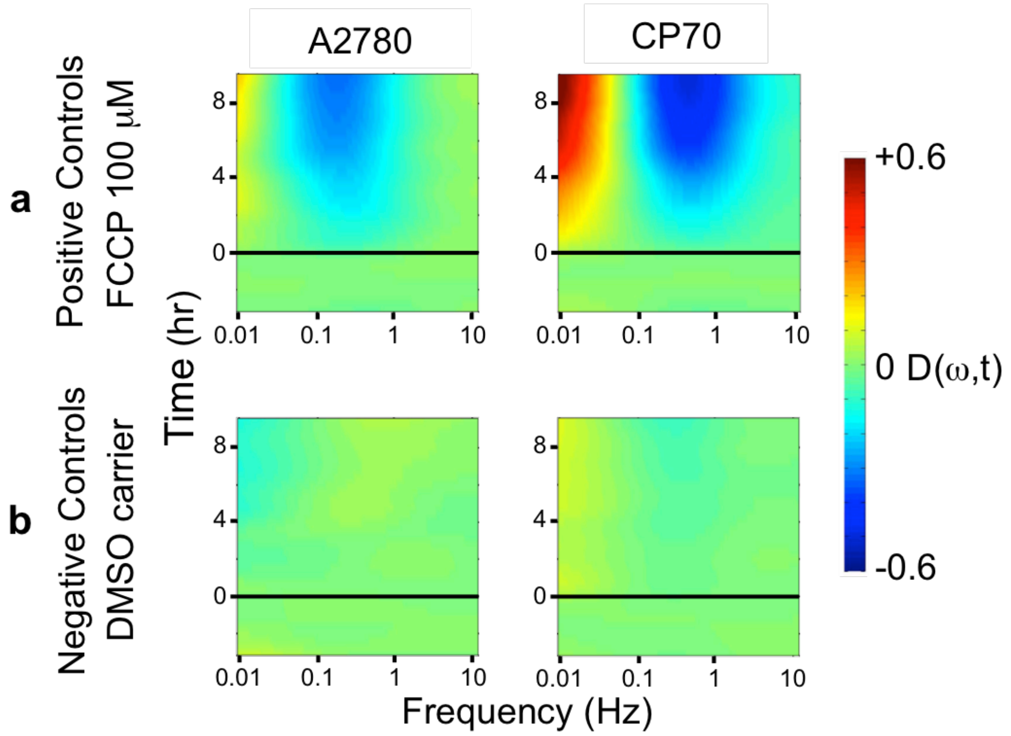

Supplementary Figure S3 Tissue dynamics spectroscopy (TDS) control experiments on tumor spheroids from A2780 and A2780/CP70 (CP70) cell lines. a) Positive control consisted of 100 μM FCCP. b) Negative controls consisted of 0.05% DMSO.

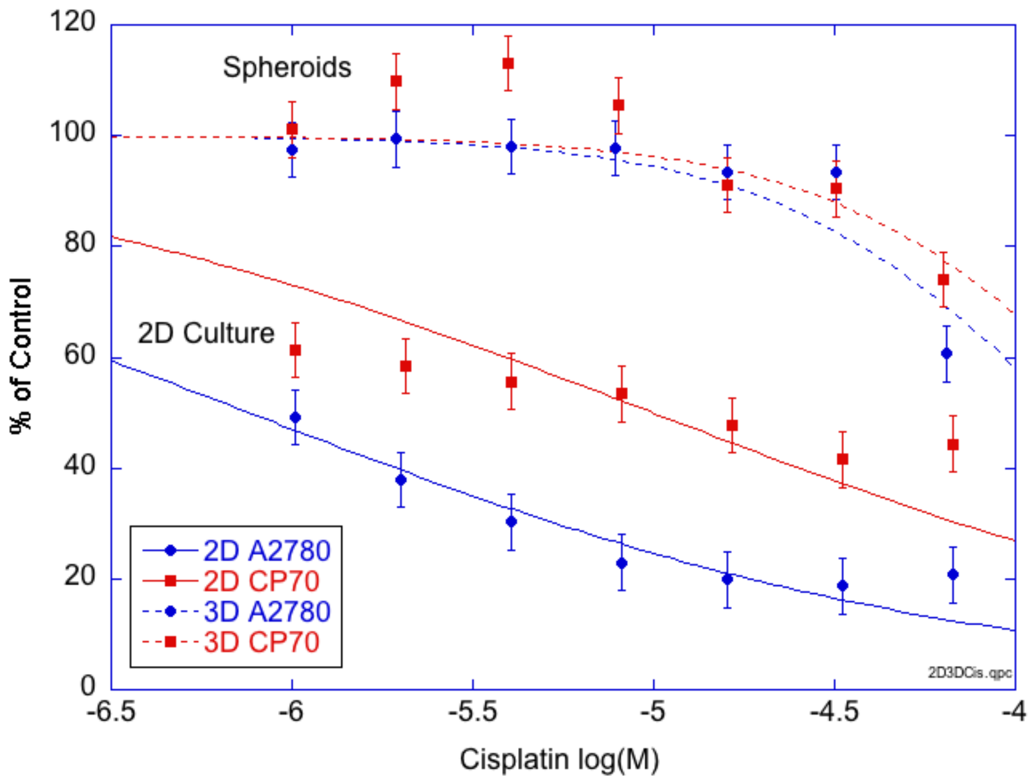

$IC50_{2DA27}$ = 1 μM $IC50_{2DCP}$ = 10 μM $IC50_{sph}$ = 160 μM

Supplementary Figure S4 IC50s over 72 hours for 2D culture compared to 3D culture (tumor spheroids) from the same cell lines. The sensitive cell line is A2780, and the insensitive cell line is A2780/CP70 (CP70). The 3D spheroids have IC50 values approximately 10 times larger than for 2D.

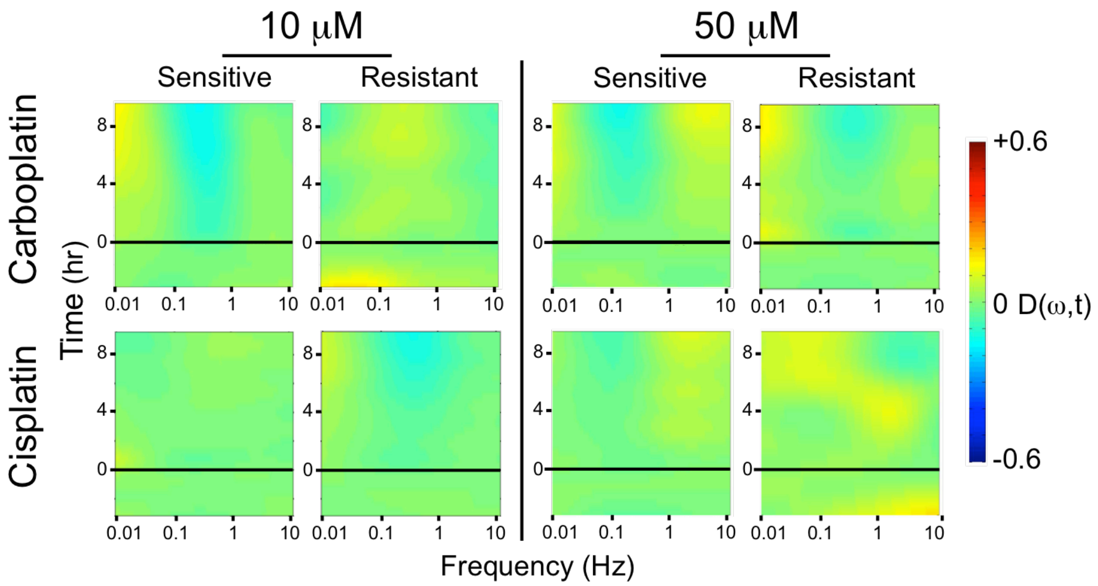

Supplementary Figure S5 Average tissue dynamics response spectrograms of spheroids grown *in vitro* for sensitive (A2780) and resistant (A2780/CP70) cell lines and both platinum compounds for 10 μm and 50 μM over 9 hours after dose. The time axis is vertical (-4 hours pre-dose to 9 hour post-dose), and the log frequency axis is horizontal (0.01 Hz to 12.5 Hz). The dose is applied at the time “0” marked by the line. The colorscale is -0.6 to 0.6 on the log difference relative to the baseline pre-dose. The IC50 for the compounds is approximately 100 μM in the 3D spheroid culture.

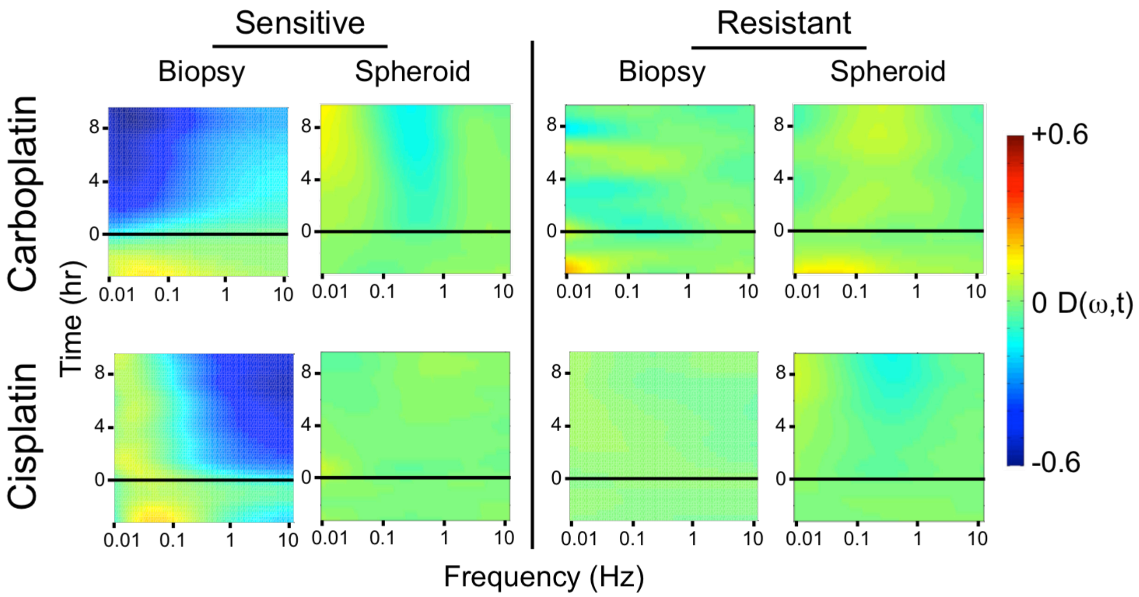

Supplementary Figure S6 Average tissue dynamics response spectrograms of sensitive/insensitive cell lines for biopsies and spheroids responding to cisplatin and carboplatin. The time axis is vertical (-4 hours pre-dose to 9 hour post-dose), and the log frequency axis is horizontal (0.01 Hz to 12.5 Hz). The dose is applied at the time marked by the line. The colorscale is -0.6 to 0.6 on the log difference relative to the baseline pre-dose. The biopsies were xenografts grown from the same cell lines as the spheroids. The resistant biopsy data to carboplatin is from a related resistant cell line (A2780cis). There is a striking difference in sensitivity between the A2780 explants relative to the spheroids.

Sample Number	ALLF	APOP	KNEE
1	-0.92	-0.45	-1.20
2	-3.29	-1.63	-1.03
3	-1.23	0.22	-1.08
4	-0.03	0.80	-1.15
5	0.15	-0.91	-0.95
6	0.51	-0.27	-0.83
7	-0.84	-0.22	-0.81
8	-1.29	-1.35	-0.64
9	-1.00	-1.16	-1.09
10	0.15	-0.91	-0.95
11	1.04	-1.07	0.40
12	0.56	0.06	0.01
13	0.53	0.38	2.13
14	0.60	0.48	1.68
15	0.81	-1.46	0.80
16	0.77	-0.02	1.04
17	-0.18	0.50	1.34
18	0.62	0.93	-0.29
19	-0.28	-0.24	0.30
20	0.56	0.44	0.47
21	0.81	1.50	-0.16
22	0.39	0.71	0.94
23	0.78	1.83	0.93
24	0.78	1.83	0.16

Supplementary Table S1 Biomarker values for each of the 24 samples used to construct the logistic predictor of drug response.
